# The Doctor’s Dilemma

**Published:** 1907-04

**Authors:** 


### "THE DOCTOR'S DILEMMA."

This is the title of a play which Bernard Shaw has written. It is being acted in London, and perhaps we shall see it here, so that a consideration of it will not be amiss. We are indebted to the British Medical Journal (Nov. 24, '06) for a sketch of the plot.

Shaw has an idea, to begin with, that many eminent physicians and surgeons have but little to do; and we all know how idleness puts one in the way of temptation. So we are prepared for the portrayal of an eminent pathologist, who is rather naughty in his way of life. This pathologist has discovered a vaccine for tuberculosis which, if used with proper regard to the opsonic index, will cure the disease. The treatment by this vaccine is still in the experimental stage; and at the time of the opening of the play it has been possible to prepare only enough for ten cases. These have been carefully selected and most of them are in hospital awaiting treatment. Now appears upon the scene an artist and his beautiful wife. At any rate she believes she is his wife; but as the artist is a complicated rascal—*a la* Shaw—she and the audience are until the end of the play left in doubt. Anyway, this very well-intentioned lady adores the artist as a man and worships him because of his artistic soul. He is dying of consumption. Will the pathologist save him? At first the latter refuses to number him among the lucky ten, but finally consents, being impressed by her representation that though her presumed husband is worthless a man, his artistic achievements are delighting and benefiting the world. Presently, however, the pathologist, finding the object of the lady's adoration to be a really impossible combination of artist and blackguard, a sort of twentieth century Benvenuto Cellini, wonders whether his life is, after all, worth saving; whether it might not after all be better for the lady that he should die before her illusions are altogether destroyed. Upon these cogitations there intrudes an old fellow-practitioner, a good man morally but of no special use to the world (he not having painted lovely and benefiting pictures), who has also developed consumption. Shall the artist be cast aside and his place among the fortunate ten be given to the inconsequential medical man. This is the "doctor's dilemma," which is further complicated when the pathologist discovers that he has fallen in love with the lady and finds that the honorable hope of marrying her in the event of the artist's death keeps bobbing up from somewhere among the sublimal strata of his consciousness. Like Banquo's ghost, this hope will not down; and he finally determines to accept for a patient his fellow-practitioner and to hand over the artist to the tender mercies of a fashionable physician whom he knows to be a wretched bungler in practice. The artist dies according to anticipation after securing the lady's promise to remarry as speedily as she can—because the idea of a sorrowing widow has always jarred upon his aesthetic sensibilities. Before the rigor mortis has set in the pathologist (who is certainly the ideal villain in the play) asks the widow for her hand, confessing that when he put the artist in the hands of the fashionable bungler, he did so in the full expectation of a fatal issue. The lady is properly shocked, as a perfect lady naturally would be, refuses him with scorn, and declares she couldn't anyway, because she has already remarried.

Several things are noteworthy about this play. For instance: There is probably no class of men so very busy, nay so overworked, as eminent physicians and surgeons. The method of preparing bacterial vaccines is at present complicated. But the basis of these vaccines is the bacteria themselves, and as, in the case of tuberculosis, the average sufferer emits several billions of baccili in a day, we would hardly fear a shortage of the essential material, nor any great difficulty in providing for more than ten cases. We hope much for these injections of bacterial vaccines; but neither they nor any other agency are going to save a case that is "dying of consumption."

"Fashionable practitioners" are not bunglers. People who live in that rather vague and mysterious realm which goes by the name of "fashionable circles" are generally well-to-do. They can afford to pay handsomely for professional services; and realizing as they do that health is by far the most desirable of all possessions, they are going to be precious careful that the physicians whom they engage are capable men.

We hope to see this play, although we haven't time to go to London for that purpose; we would certainly find it diverting in ways not intended by its author.

